# Toward population specific and personalized treatment of *Helicobacter pylori* infection

**DOI:** 10.1186/s12929-018-0471-z

**Published:** 2018-10-02

**Authors:** Jyh-Ming Liou, Po-Yueh Chen, Yu-Ting Kuo, Ming-Shiang Wu, for the Taiwan Gastrointestinal Disease and Helicobacter Consortium, Jyh-Ming Liou, Yi-Chia Lee, Mei-Jyh Chen, Jaw-Town Lin, Chun-Ying Wu, Jeng-Yih Wu, Ching-Chow Chen, Chun-Hung Lin, Yu-Ren Fang, Ming-Jong Bair, Jiing-Chyuan Luo, Ming-Shiang Wu, Tsu-Yao Cheng, Ping-Huei Tseng, Han-Mo Chiu, Chun-Chao Chang, Chien-Chun Yu, Min-Chin Chiu, Yen-Nien Chen, Wen-Hao Hu, Chu-Kuang Chou, Chi-Ming Tai, Ching-Tai Lee, Wen-Lun Wang, Wen-Shiung Chang

**Affiliations:** 1 Division of Gastroenterology and Hepatology, Department of Internal Medicine, National Taiwan University Hospital, Taipei, Taiwan; 2 Department of Internal Medicine, College of Medicine, National Taiwan University, No. 7, Chung-Shan S. Road, Taipei, Taiwan; 3 Division of Gastroenterology and Hepatology, Department of Internal medicine, Chia-Yi Christian Hospital, Chia-Yi, Taiwan

**Keywords:** *H. pylori*, Resistance, Eradication, First-line, Rescue, Precision medicine, Gastric cancer

## Abstract

In the face of rising prevalence of antibiotic resistance, susceptibility testing to provide personalized treatment is recommended prior to eradication therapy for *Helicobacter pylori* (*H. pylori)*. Yet, population specific treatment according to the local prevalence of antibiotic resistance may be an alternative if susceptibility testing is not available. In this article, we reviewed the global prevalence of primary antibiotic resistance and the efficacies of commonly used regimens in antibiotic susceptible and resistance strains. We then constructed a model to predict the efficacies of these regimens and proposed an algorithm to choose the optimal first-line and rescue therapies according to the prevalence of antibiotic resistance. Clarithromycin-based therapy (triple, sequential, concomitant, and hybrid therapies) for 14 days remains the treatment of choice in regions with low clarithromycin resistance (≤15%) and bismuth quadruple therapy may be an alternative therapy. In regions with high clarithromycin resistance (> 15%), bismuth quadruple therapy is the treatment of choice and non-bismuth quadruple therapy may be an alternative. Either levofloxacin-based therapy or bismuth quadruple therapy may be used as second-line rescue therapy for patients fail after clarithromycin-based therapies, whereas levofloxacin-based therapy may be used for patients fail after bismuth quadruple therapy. Susceptibility testing or genotypic resistance should be determined after two or more eradication failures. However, empirical therapy according to prior medication history to avoid the empirical reuse of levofloxacin and clarithromycin may be an acceptable alternative after consideration of cost, patient preference, and accessibility. Rifabutin-based therapy for 14 days may serve as the fourth-line therapy. New antibiotics specific for *H. pylori* are highly anticipated.

**Electronic supplementary material:**

The online version of this article (10.1186/s12929-018-0471-z) contains supplementary material, which is available to authorized users.

## Background

*Helicobacter pylori (H. pylori)* infection is a causal factor of peptic ulcer disease, gastric cancer (adenocarcinoma) and mucosal associated lymphoid tissue lymphoma [1]. Eradication of *H. pylori* may reduce the recurrence rate of peptic ulcer and may reduce the risk of gastric cancer [1–3]. However, the efficacy of standard triple therapy containing a proton pump inhibitor (PPI), clarithromycin, with amoxicillin or metronidazole has been declining in many countries [4, 5]. Factors that might lead to treatment failure include the presence of antibiotic resistance, lack of good compliance, inadequate treatment length, and inadequate suppression of gastric acid secretion [6, 7]. Of these, the presence of antibiotic resistance is the most important factor [6, 7]. Therefore, the best strategy to increase the eradication rate is to provide individualized treatment according to antibiotic susceptibility testing (personalized treatment) [8]. However, endoscopy with biopsy and culture for *H. pylori* are costly and time consuming (2–4 weeks). Besides, the successful rate of culture and susceptibility testing ranges from 75 to 90% [9, 10]. Therefore, susceptibility testing guided therapy is not widely applicable for the first-line therapy and is not easily accessible even for refractory *H. pylori* infection [11, 12]. Development of less invasive and less costly tests, such as genotyping of antibiotic resistance genes using gastric biopsy, gastric juice or fecal samples might be an alternative [10]. Yet, the accuracies of these tests using fecal samples are still less than perfect. Another strategy is to choose the best regimen for a population according to the prevalence of antibiotic resistance (population specific treatment) [13–16]. The efficacy of a regimen for *H. pylori* eradication can be predicted as long as its efficacies in susceptible and resistant strains and the prevalence of antibiotic resistance in the population are known [17, 18]. Therefore, we reviewed the global prevalence of antibiotic resistance and the efficacies of different regimens in antibiotic susceptible and resistant strains and constructed prediction models to predict the efficacies of these regimens in regions with different prevalence of antibiotic resistance in this article. Finally, we proposed an algorithm to choose the optimal first-line and rescue therapies according to the prevalence of antibiotic resistance.

## Updated prevalence of primary antibiotic resistance worldwide [19–24]

**Fig. 1:**
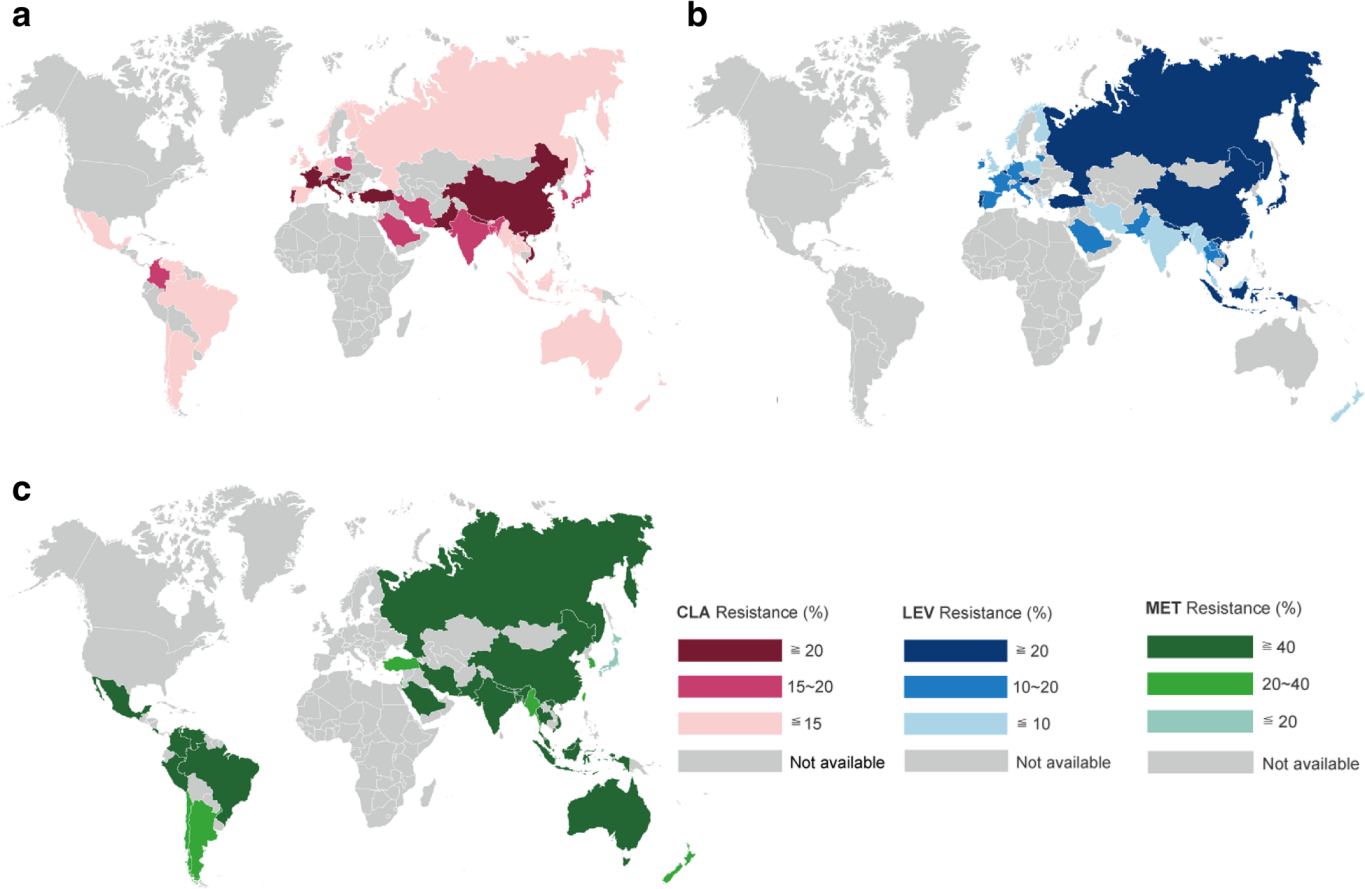
Updated prevalence of (**a**) clarithromycin, (**b**) levofloxacin, and (**c**) metronidazole resistance of *Helicobacter pylori*. CLA: clarithromycin; LEV: levofloxacin; MET: metronidazole

The prevalence of primary antibiotic resistance varies from country to country and changes with time. The updated global prevalence of antibiotic resistance was as follows (Fig. 1).

### Clarithromycin resistance

The overall prevalence of primary clarithromycin resistance was 10% (95% CI 4–16) in America’s region [22], 17% (95% CI 15–18) in Asia-Pacific [5], and 18% (95% CI 16–20) in Europe [22]. However, there were trends of rising clarithromycin resistance in these regions. The pooled resistance rates of clarithromycin resistance after 2011 were 21% (95% CI 18–25%) in Asia-Pacific, 20% (95% CI 12–28%) in America, and 28% (95% CI 25–31%) in Europe, as shown in Table 1. In Asia-Pacific region [5], clarithromycin resistance was higher than 15% in 13 countries: Bangladesh, China, India, Iran, Japan, Nepal, New Zealand, Pakistan, Saudi Arabia, Singapore, South Korea, Turkey, and Vietnam. In contrast, frequency of resistance was less than 15% in eight countries: Bhutan, Indonesia, Laos, Malaysia, Myanmar, Russia (data were specifically from eastern Russia), Taiwan, and Thailand (Fig. 1).

**Table 1:** Prevalence of primary antibiotic resistance of *H pylori* by time period, stratified by WHO region

WHO region	Prevalence of primary resistance		
	Clarithromycin	Metronidazole	Levofloxacin
Americas region [23]			
2006–2008	11 (3–19)	26 (10–42)	N/A
2009–2011	9 (2–15)	21 (13–33)	11 (5–16)
2012–2016	20 (12–28)	29 (0–59)	19 (5–16)
European region [23]			
2006–2008	28 (24–32)	38 (33–43)	15 (12–18)
2009–2011	23 (20–27)	33 (25–40)	13 (9–17)
2012–2016	28 (25–31)	46 (34–58)	12 (8–15)
Asia-Pacific region [5]			
2006–2010	19 (16–23)	50 (44–56)	17 (13–21)
2011–2015	21 (18–25)	45 (39–48)	27 (21–34)

*WHO* world health organization

### Metronidazole resistance

The overall prevalence of primary clarithromycin resistance was 23% (95% CI 2–44) in Americas [22], 32% (95% CI 27–36) in Europe [22], and 44% (95% CI 39–48) in Asia-Pacific [5]. Although there were no remarkable changes in metronidazole resistance over time compared to clarithromycin, the pooled prevalence of primary metronidazole resistance after 2011 was greater 25% in these regions (Table 1). According to data for 2006–15 in Asia-Pacific, metronidazole resistance was higher than 40% in most countries, except Japan, Myanmar, South Korea, Taiwan, and Turkey [5].

### Levofloxacin resistance

The overall prevalence of primary levofloxacin resistance was 11% (95% CI 9–13) in Europe [22], 15% (95% CI 5–16) in Americas [22], and 18% (95% CI 15–22) in Asia-Pacific [5]. Prevalence of resistance to levofloxacin in America and Asia-Pacific rose significantly over time during the period investigated. The pooled prevalence of primary levofloxacin resistance after 2011 was 19% (95% CI 5–16%) in America, 12% (95% CI 8–15%) in Europe, and 27% (95% CI 21–34%) in Asia-Pacific (Table 1). In Asia-Pacific regions, resistance to levofloxacin increased over time in all included countries, except in Iran. The levofloxacin resistance rates were significantly higher in Eastern Asia (including China, Hong Kong, Japan, South Korea, and Taiwan) than in western Asia (including Israel, Saudi Arabia, and Turkey) and southeastern Asia (including Indonesia, Laos, Malaysia, Myanmar, Singapore, Thailand, and Vietnam) [5]. Megraud et al. [19] and Liou et al. [21] showed that fluoroquinolone resistance correlated with consumption of fluoroquinolones in Europe and Taiwan, respectively. The global consumption of fluoroquinolones has significantly increased since 2000 [23], which might be explained by the recommendation in 2004 guidelines to use fluoroquinolone monotherapy as an alternative first-line therapy for community-acquired pneumonia [24].

### Amoxicillin resistance

The overall prevalence of primary amoxicillin resistance was 0% (95% CI 0–0) in Europe [22], 3% (95% CI 2–4) in Asia-Pacific [5], and 10% (95% CI 2–19) in Americas [22]. The trend in amoxicillin resistance was only available in Asia-Pacific region and country-specific data showed no remarkable changes in resistance over time [5]. Although amoxicillin resistance was uncommon in the Asia-Pacific region, resistance to amoxicillin was higher than 10% in Pakistan and India.

### Tetracycline resistance

The overall prevalence of primary tetracycline resistance was 0% (95% CI 0–0) in Europe, [23] 4% (95% CI 2–5) in Asia-Pacific [5], and 4% (95% CI 1–11) in Americas [22]. The trend in tetracycline resistance was only available in Asia-Pacific region and no remarkable changes over time [5]. The prevalence of resistance to tetracycline was < 10% in all countries, except Pakistan and India, where tetracycline resistance was higher than 10%.

## Strategies to improve the efficacy of first-line therapy

The dosages and frequencies of PPI, bismuth, and antibiotics of the commonly used regimens are shown in Table 2. There are several strategies to improve the efficacy of first-line therapy, including extending the length of treatment to 14 days, the use of vonoprazan or higher dosage of PPI, the use of four drug regimens (bismuth quadruple therapy, concomitant therapy, sequential therapy, or hybrid therapy), susceptibility testing (or genotypic resistance) guided therapy, and supplementation with probiotics (Table 3) [25–39].

**Table 2:** Regimens commonly used for *H. pylori* eradication

First-line Regimens	Dosing and frequencies
Clarithromycin triple therapy	A PPI bid, clarithromycin 500 mg bid, and amoxicillin 1 g bid or metronidazole 500 mg bid for 7–14 days
Bismuth quadruple therapy	A PPI bid, bismuth qid, tetracycline 500 mg qid, and metronidazole 500 mg tid for 7–14 days
Sequential therapy	A PPI bid plus amoxicillin 500 mg bid for 5–7 days, followed by a PPI bid plus clarithromycin 500 mg bid and metronidazole 500 mg bid for another 5–7 days
Concomitant therapy	A PPI bid plus amoxicillin 500 mg bid, clarithromycin 500 mg bid and metronidazole 500 mg bid for 7–14 days
Hybrid therapy	A PPI bid plus amoxicillin 500 mg bid for 5–7 days, followed by a PPI bid plus amoxicillin 500 mg bid, clarithromycin 500 mg bid and metronidazole 500 mg bid for another 5–7 days
Second-line/ third regimens	
Levofloxacin triple therapy	A PPI bid, levofloxacin 500 mg qd, and amoxicillin 1 g bid for 10–14 days
Levofloxacin sequential therapy	A PPI bid plus amoxicillin 500 mg bid for 7 days, followed by a PPI bid plus levofloxacin 250 mg bid and metronidazole 500 mg bid for another 7 days
Levofloxacin concomitant therapy	A PPI bid plus amoxicillin 500 mg bid, levofloxacin 250 mg bid and metronidazole 500 mg bid for 7–14 days
Bismuth quadruple therapy	A PPI bid, bismuth qid, tetracycline 500 mg qid, and metronidazole 500 mg tid for 7–14 days
Fourth-line regimen	
Rifabutin triple therapy	A PPI bid, rifabutin 150 mg bid, and amoxicillin 1 g bid for 14 days

Dosage of proton pump inhibitors (PPI): omeprazole 20 mg, lansoprazole 30 mg, esomeprazole 20 mg, pantoprazole 40 mg, rabeprazole 20 mg

**Table 3:** Strategies to improve the efficacy of first-line therapy

Strategy for improvement	Supporting evidence
Extending the treatment length of triple therapy to 14 days	Meta-analysis of 59 randomized trials showed that triple therapy for 14 days is more effective than triple therapy given for 7 or 10 days [26].
Use of higher dosage of PPI or vonoprazan	Meta-analysis of 6 randomized trials showed that the use of higher dosage of PPI may increase the eradication rate. Two randomized trials showed that vonoprazan-based triple therapy was superior to standard dose PPI-based triple therapy, particularly for clarithromycin resistant strains [30–32].
Use of four drug regimen	
Bismuth quadruple therapy	Randomized trials showed that bismuth quadruple therapy was superior to triple therapy in regions with high clarithromycin resistance (> 15%) [29, 33, 35].
Concomitant therapy	Meta-analysis of randomized trials showed that concomitant therapy given for 5 or 10 days was superior to 5- or 7- or 10-day PAC based triple therapy, but was not superior to 14-day triple therapy. A non-randomized trial showed that 14-day concomitant therapy was superior to 14-day triple therapy [29, 34, 38, 39].
Sequential therapy	Meta-analysis of randomized trials showed that 10-day sequential therapy was superior to triple therapy for 10 days or less, but was not superior to 14-day triple therapy. Meta-analysis of 4 randomized trials showed that 14-day sequential therapy was superior to 14-day triple therapy [27, 28, 33].
Hybrid therapy	A randomized trial showed that 14-day hybrid therapy was superior to 14-day triple therapy. Another randomized trial showed that 12-day reverse hybrid therapy was superior to 12-day triple therapy [37].
Susceptibility testing guided therapy	Meta-analysis of randomized trials showed that susceptibility testing guided therapy was superior to empirical triple therapy given for 7 or 10 days [8].
Supplementation with probiotics	Meta-analysis of randomized trials showed that supplementation with probiotics may reduce the adverse effects and increase the efficacy of triple therapy [40–43].

*PPI* proton pump inhibitor

### Extending the treatment length of triple therapy to 14 days

Clarithromycin-based triple therapy remains one of the treatment options in countries where the prevalence of clarithromycin resistance is lower than 15% [13–16, 25]. A Cochrane meta-analysis of 59 randomized trials showed that the efficacy of triple therapy may be increased by extending its treatment length from 7 days to 10 days (75.7% vs 79.9%, RR 0.80, 95% CI 0.72 to 0.89), from 7 or 14 days (72.9% vs 81.9%, RR 0.66, 95% CI 0.60 to 0.74), or from 10 days to 14 days (78.5% vs 84.4%, RR 0.72, 95% CI 0.58 to 0.90) [26]. Therefore, extending the treatment length of triple therapy to 14 days is recommended in several international consensus reports [13–16, 25].

### Use of higher dosage of PPI or vonoprazan

The minimum inhibitory concentrations (MICs) of amoxicillin, clarithromycin, and levofloxacin are higher in acidic environment [7, 9]. Therefore, increasing the gastric pH values through the use of higher dosage of PPI may increase the efficacy of eradication therapy for *H. pylori* [7]. The standard dosages of PPI used for *H. pylori* eradication were omeprazole 20 mg, esomeprazole 20 mg, pantoprazole 40 mg, lansoprazole 30 mg, and rabeprazole 20 mg given twice daily. Meta-analysis of 6 randomized trials (*N* = 1703) showed that the use of higher dosage of PPI may increase the eradication rate of standard triple therapy [30, 31]. However, only two trials compared the same PPI of different dosage [30, 31]. Vonoprazan, a potassium-competitive acid blocker (P-CAB), is a novel gastric acid secretion suppressant. A randomized trial showed that vonoprazan-based triple therapy is superior to lansoprazole-based triple therapy in Japan, especially for clarithromycin resistant strains [32]. It’s efficacy against clarithromycin resistant strains has been confirmed in several retrospective or prospective non-randomized studies in Japan. However, the finding needs to be validated in more trials outside Japan.

### Use of four drug regimen

Clarithromycin based triple therapy is not recommended in countries where the prevalence of clarithromycin resistance is higher than 15% in international consensus reports [13–16, 25]. Bismuth quadruple therapy or non-bismuth quadruple therapies (concomitant therapy, sequential therapy, hybrid therapy) are recommended in these regions [13–16, 25, 27–29, 33–37]. Recent meta-analysis of randomized trials showed that 14-day sequential therapy, but not 10-day sequential therapy, was superior to 14-day triple therapy [13]. A recent randomized trial showed that 14-day sequential therapy was not inferior to 10-day bismuth quadruple therapy [33]. Therefore, extending the treatment length of sequential therapy to 14 days is recommended [27–29, 33]. Our recent systematic review and meta-analysis showed that concomitant therapy for 5, 7 or 10 days was superior to triple therapy for 7 or 10 days, but was not superior to 14-day triple therapy [38]. A non-randomized trial showed that 14-day concomitant therapy was superior to 14-day triple therapy [39]. Therefore, the treatment length of concomitant therapy is 14 days in several international consensus reports [13–16]. Although the Maastricht V and the Toronto Consensus recommended that bismuth quadruple therapy should be given for 14 days, the evidence level supporting the recommendation is low [13, 14]. Our recent trials showed that bismuth quadruple therapy given for 10 days was superior to 14-day triple therapy and its efficacy was greater than 90% in Taiwan [36]. Therefore, 10-day bismuth quadruple therapy is an acceptable regimen in Taiwan.

### Susceptibility testing guided therapy

Meta-analysis of 9 randomized trials including 1958 subjects showed that susceptibility testing guided therapy was more effective than empirical triple therapy for 7 or 10 days in the first-line treatment of *H. pylori* infection [8]. However, most of these trials randomize patients after endoscopy and/or culture which is not similar to that in clinical practice because patients might decline endoscopy, the yield rate of culture is only 70–90%, and the accuracy of susceptibility testing is not 100% [8]. Besides, whether susceptibility testing guided therapy is superior to 14-day triple therapy or bismuth quadruple therapy are still unknown.

### Supplementation with probiotics

A recent meta-analysis showed that probiotics may induce a significant reduction in delta values of urea breath test than placebo (8.61% with a 95%CI: 5.88–11.34, vs 0.19% for placebo, *P* < 0.001) [40]. However, only about 10–15% of *H. pylori* infection was eradicated with probiotic monotherapy [40]. Earlier studies showed that supplementation of probiotics may increase the eradication rate of triple therapy, probably through the alleviation of adverse effects of triple therapy [41]. However, more recent meta-analysis of 21 randomized control trials showed that standard therapy plus probiotics may reduce the frequency of adverse effect compared to standard therapy with or without a placebo, but does not increase the eradication rate of standard therapy [42]. Yet, another meta-analysis of randomized trial showed that adjunctive use of some multi-strain probiotics may increase the eradication rate and reduce the risk of adverse events but not all mixtures were effective [43]. Therefore, routine supplementation of probiotics is not recommended in the Toronto and the Asean Consensus Reports considering the controversial results and the cost [14, 15].

## Efficacies of different eradication regimens in susceptible and resistant strains

The efficacies of six commonly used regimens in susceptible and resistant strains in the first-line treatment of *H. pylori* infection were reviewed in this article. Pooled analyses of efficacies of the six different regimens in antibiotic susceptible and resistant strains according to the length of treatment were shown in Table 4 and in Additional file 1: Tables S1-S6 [8–30, 33–38]. Except for 5-day concomitant therapy and 7-day bismuth quadruple therapy, the eradication rates of the other regimens were greater than 90% in clarithromycin susceptible strains (Table 4). However, the efficacy of levofloxacin triple therapy was only 87.5% in the first-line therapy, even for levofloxacin susceptible strains. The efficacies of triple therapy, sequential therapy, concomitant therapy, and hybrid therapy were significantly lower in clarithromycin resistant strains, especially when the treatment length were 10 days or less (Table 4). The efficacies of bismuth quadruple therapy were not affected by clarithromycin resistance. However, the efficacy of bismuth quadruple therapy was affected by metronidazole resistance when it was given for 7 days.

**Table 4:** Eradication rate in susceptible and resistant strains^a^[8–30, 33–38]

	Clarithromycin susceptible	Clarithromycin resistant
Triple therapy: PPI-amoxicillin-clarithromycin		
7 days	88.5% (2428/2744)	25.8% (121/469)
10 days	90.8% (267/294)	44% (37/84)
14 days	89.6% (841/939)	43.3% (55/127)
Sequential therapy		
10 days	91% (1470/1616)	65% (225/346)
14 days	98.1% (304/310)	72.2% (26/36)
Concomitant therapy		
5 days	84.4% (76/90)	50% (2/4)
7 days	96.3% (181/188)	83.3% (20/24)
10 days	94.5% (598/633)	80.5% (120/149)
Hybrid therapy		
10–14 days	96.8% (418/432)	81.8% (117/143)
Bismuth quadruple therapy		
7 days	87.2% (321/368)	87.2% (321/368)
10 days	93.9% (512/545)	91.4% (139/152)
14 days	96.9% (94/97)	92.3% (12/13)
Bismuth quadruple therapy	Metronidazole susceptible	Metronidazole resistant
7 days	92% (252/274)	73.4% (69/94)
10 days	94.3% (764/810)	89.8% (397/442)
14 days	96.1% (99/103)	93.2% (41/44)
	Levofloxacin susceptible	Levofloxacin resistant
Triple therapy: PPI-amoxicillin-levofloxacin	81.8% (189/231)	33.3% (10/30)

*PPI* proton pump inhibitor

^a^detailed data shown in supplementary materials

## Prediction of different regimens in regions with different prevalence of antibiotic resistance

**Fig. 2:**
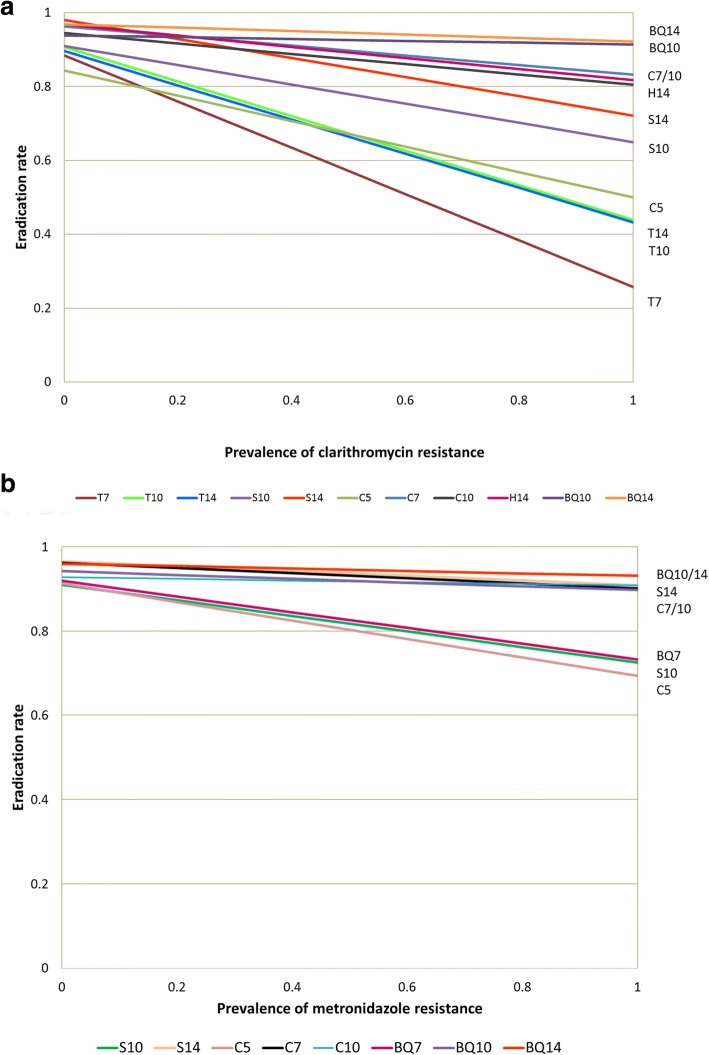
Predicted efficacies of different regimens according to prevalence of (**a**) clarithromycin resistance and (**b**) metronidazole resistance. T7: triple therapy for 7 days; T10: triple therapy for 10 days; T14: triple therapy for 14 days; S10: sequential therapy for 10 days; S14: sequential therapy for 14 days; C5: concomitant therapy for 5 days; C7: concomitant therapy for 7 days; C10: concomitant therapy for 10 days; H14: hybrid therapy for 14 days; BQ10: bismuth quadruple therapy for 10 days; BQ14: bismuth quadruple therapy for 14 days

The efficacy of a regimen which contains antibiotic A (drug A) and antibiotic B (drug B) in a region can be predicted if the prevalence of antibiotic resistance in that region and the efficacy of this regimen in susceptible and resistant strains are known [17, 18] . Assuming the prevalence of antibiotic resistance for drug A and drug B are p and q, respectively, the prevalence of dual drug resistance and dual susceptible strains would be p*q and (1-p)*(1-q), respectively. Therefore, the estimated eradication rate of that regimen would be 【ER_SS_* (1-p)*(1-q)】 + 【ER_SR_* (1-p)*q】 + 【ER_RS_ *P*(1-q)】 + 【ER_RR_* P*q】, where ER_SS,_ ER_SR,_ ER_RS_, and ER_RR_ are eradication rates in dual susceptible, susceptible to drug A but resistant to drug B, resistant to drug A but susceptible to drug B, and dual resistant strains, respectively. Based on this prediction model and the efficacies of different regimens in antibiotic susceptible and resistant strains, the efficacies of these regimens in regions with different prevalence of antibiotic resistance can be predicted, as shown in Fig. 2. For example, the predicted efficacy of 7-day standard triple therapy according to the prevalence of clarithromycin resistance would be 0.885(1-p) + 0.258p (p is the prevalence of clarithromycin resistance). Comparing to other regimens, the eradication rates of 7-day, 10-day, 14-day triple therapy and 5-day concomitant therapy would be lower than 80% in regions where the prevalence of clarithromycin resistance is higher than 20% (Fig. 2). Among these regimens, the efficacy of bismuth quadruple therapy would remain higher than 90% in regions with high prevalence of primary clarithromycin resistance (Fig. 2). The efficacies of metronidazole-containing regimens, including sequential therapy, concomitant therapy, hybrid therapy and bismuth quadruple therapy were also affected by metronidazole resistance, but the effect size was relatively smaller (Fig. 2). The efficacy of levofloxacin triple therapy for treatment-naïve patients would be lower than 80% when the levofloxacin-resistant rate higher than 15%.

Based on the Hp-normogram in Fig. 2, bismuth quadruple therapy and non-bismuth quadruple therapy (14-day sequential therapy, 14-day concomitant therapy, and 14-day hybrid therapy) are the preferred regimens for the first-line treatment of *H. pylori* infection in regions with higher prevalence of clarithromycin resistance. Standard triple therapy given for 14 day may still be an option in regions where the prevalence of clarithromycin resistance is lower than 15%. Levofloxacin triple therapy is not recommended in the first- line treatment of *H. pylori* infection due to its low efficacy.

## Second-line therapy

**Fig. 3:**
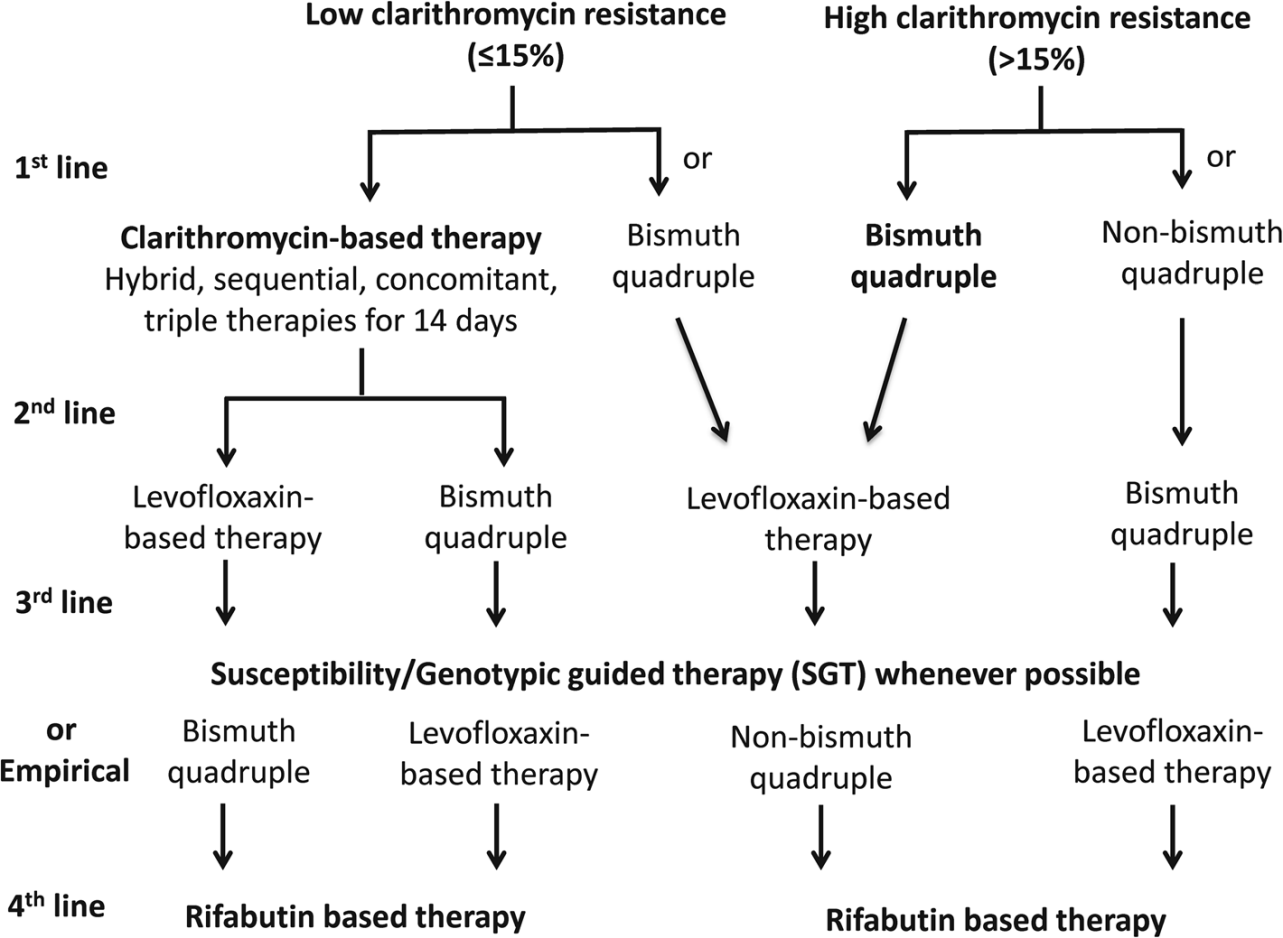
Recommended algorithm for population specific treatments

After failure of one eradication therapy, the choice of second-line eradication regimen can be empirical or guided by susceptibility testing [13–16, 25]. A recent meta-analysis of 4 randomized trials failed to show the superiority of susceptibility testing guided therapy over empirical therapy in the second-line therapy, probably attributed to the small sample size and the heterogeneity among the trials [8]. Therefore, the majority of these patients were treated empirically in clinical practice. Antibiotics used in previous eradication therapy are important and helpful to guide the second-line rescue therapy (Fig. 3). The Taiwan Consensus Report recommended the avoidance of empirical reuse of clarithromycin and levofloxacin without susceptibility testing because the secondary resistance rates of clarithromycin and levofloxacin are high for patients who fail after clarithromycin-based and levofloxacin-based therapies, respectively [25]. Bismuth quadruple therapy and levofloxacin based therapy are the most commonly used second-line rescue regimens for patients who fail after clarithromycin-based therapies [13–16, 25]. An earlier systematic review and meta-analysis showed similar efficacies of levofloxacin triple therapy and bismuth quadruple therapy in the second-line therapy [44]. However, the frequency of adverse effects was higher for bismuth quadruple therapy than levofloxacin triple therapy [44]. Yet, the prevalence of levofloxacin resistance is rising in recent years in many parts of the world [5, 19–22]. Therefore, Chen et al. found that the efficacy of levofloxacin triple therapy was only 74% in the second-line therapy in a recent systematic review and meta-analysis [45]. Liou et al. further showed that levofloxacin sequential therapy for 10 days was superior to levofloxacin triple therapy for 10 days in the second-line treatment in Taiwan [46, 47]. Levofloxacin concomitant therapy given for 5 days has been shown to be similarly effective as levofloxacin sequential therapy for 10 days in the first-line therapy, but its efficacy in the second-line therapy remains unknown [48]. In another randomized trial in Taiwan, Hsu et al. showed that modified bismuth quadruple therapy containing bismuth, a PPI, tetracycline, and levofloxacin for 10 days was superior to levofloxacin triple therapy for 10 days in the second-line therapy [49]. Non-bismuth quadruple therapy (preferably concomitant therapy) may be a second-line rescue therapy for patients who fail after bismuth quadruple therapy, but the level of evidence is low for this recommendation [13–16].

## Treatment of refractory *H. pylori* infection

Refractory *H. pylori* infection is defined as failure after two or more eradication therapies. Earlier Maastricht Consensus Reports recommended that susceptibility testing should be done after failure of two eradication therapies whenever possible [50] . However, susceptibility testing for *H. pylori* is not widely available because of it is costly (endoscopy required), time consuming (2–4 weeks) and the successful culture rate varies from 70 to 90%. Besides, the reported efficacies of susceptibility testing guided therapy were not satisfactory, ranging from 36 to 91% in some published retrospective or prospective case series [11, 12]. Therefore, the majority of patients are treated empirically in routine clinical practice. Bismuth quadruple therapy and levofloxacin-based therapy are commonly used as third-line rescue therapy, whereas rifabutin-based therapy is usually reserved as fourth-line rescue therapy [13–16, 25]. Bismuth quadruple therapy may be used as the third-line rescue therapy for patients fail after clarithromycin-based therapy and levofloxacin-based therapy in previous eradication therapies [13–16]. Levofloxacin-based therapy may be used as the third-line rescue therapy for patients fail after clarithromycin-based therapy and bismuth quadruple therapy. 23S rRNA mutations and gyrase A mutations correlate well with clarithromycin and levofloxacin resistance, respectively [10]. Our previous pilot trial showed that genotypic resistance guided therapy may achieve 80% eradication rate in the third line treatment [51]. Therefore, we further conducted a multicenter randomized trial to compare the efficacies of genotypic resistance guided therapy vs. empirical therapy for refractory *H. pylori* infection [52]. We found that *H. pylori* was eradicated in 160/205 patients receiving genotypic resistance-guided therapy (78%) and 148/205 patients receiving empirical therapy 72.2% (*P* = 0.170) [52]. This is the first randomized trial to show that properly designed empirical therapy is an acceptable alternative to genotypic resistance-guided therapy for eradication of refractory *H. pylori* infection after consideration of cost, patient preference, and accessibility [52]. However, further studies are warranted to compare the efficacy of susceptibility testing guided therapy to genotypic resistance guided therapy or empirical therapy according to medication history.

## Conclusion

The rising prevalence of primary clarithromycin and levofloxacin resistance of *H. pylori* is a global problem. However, the prevalence of antibiotic resistance varies greatly in different countries and regions. We proposed an algorithm to choose the optimal first-line and rescue therapies according to the prevalence of antibiotic resistance in this article (Fig. 3). Clarithromycin-based therapy (triple, sequential, concomitant, and hybrid therapies) given for 14 days remains the treatment of choice in regions with low clarithromycin resistance (≤15%). Bismuth quadruple therapy may be an alternative therapy in this region. In regions with high clarithromycin resistance (> 15%), bismuth quadruple therapy is the treatment of choice. Non-bismuth quadruple therapy may be an alternative if the prevalence of dual clarithromycin and metronidazole resistance is lower than 10%. Either levofloxacin-based therapy or bismuth quadruple therapy may be used as second-line rescue therapy for patients fail after clarithromycin-based therapies, whereas levofloxacin-based therapy may be used for patients fail after bismuth quadruple therapy. Susceptibility testing or genotypic resistance should be determined after two or more eradication failures. However, empirical therapy according to prior medication history to avoid the empirical reuse of levofloxacin and clarithromycin may be an acceptable alternative after consideration of cost, patient preference, and accessibility. Rifabutin-based therapy given for 14 days may be used as the fourth-line rescue therapy. New antibiotics specific for *H. pylori* are highly anticipated.

## Additional file

Additional file 1: **Table S1–1**. Efficacy of 7-day triple therapy in the first line treatment of the individual studies. **Table S1–2**. Efficacy of 10-day triple therapy in the first line treatment of the individual studies. **Table S1–3**. Efficacy of 14-day triple therapy in the first line treatment of the individual studies. **Table S2–1**. Efficacy of 10-day sequential therapy in the first line treatment of the individual studies. **Table S2–2**. Efficacy of 14-day sequential therapy in the first line treatment of the individual studies. **Table S3–1**. Efficacy of 5-day or less concomitant therapy in the first line treatment of the individual studies. **Table S3–2**. Efficacy of 7-day concomitant therapy in the first line treatment of the individual studies. **Table S3–3**. Efficacy of 10-day concomitant therapy in the first line treatment of the individual studies. **Table S3–4**. Efficacy of 14-day concomitant therapy in the first line treatment of the individual studies. **Table S4**. Efficacy of 10–14 day hybrid therapy in the first line treatment of the individual studies. **Table S5–1**. Efficacy of 7-day or less bismuth quadruple therapy in the first line treatment of the individual studies. **Table S5–2**. Efficacy of 10-day bismuth quadruple therapy in the first line treatment of the individual studies. **Table S5–3**. Efficacy of 14-day bismuth quadruple therapy in the first line treatment of the individual studies. **Table S6**. Efficacy of Levofloxacin triple therapy in the first line treatment of the individual studies. (DOCX 221 kb)

## Abbreviations

BQ10: Bismuth quadruple therapy for 10 days
BQ14: Bismuth quadruple therapy for 14 days
C10: Concomitant therapy for 10 days
C5: Concomitant therapy for 5 days
C7: Concomitant therapy for 7 days
CIs: Confidence intervals
CLA: Clarithromycin
*H. pylori*: *Helicobacter pylori*
H14: Hybrid therapy for 14 days
LEV: Levofloxacin
MET: Metronidazole
PPI: Proton pump inhibitor
S10: Sequential therapy for 10 days
S14: Sequential therapy for 14 days
T10: Triple therapy for 10 days
T14: Triple therapy for 14 days.
T7: Triple therapy for 7 days
