# Supplementary material for: Toward population specific and personalized treatment of Helicobacter pylori infection
Source: J Biomed Sci. 2018 Oct 2;25:70. doi: 10.1186/s12929-018-0471-z (PMC6167866; doi:10.1186/s12929-018-0471-z)
Supplement: Supplementary file 1 — Table S1–1. Efficacy of 7-day triple therapy in the first line treatment of the individual studies. Table S1–2. Efficacy of 10-day triple therapy in the first line treatment of the individual studies. Table S1–3. Efficacy of 14-day triple therapy in the first line treatment of the individual studies. Table S2–1. Efficacy of 10-day sequential therapy in the first line treatment of the individual studies. Table S2–2. Efficacy of 14-day sequential therapy in the first line treatment of the individual studies. Table S3–1. Efficacy of 5-day or less concomitant therapy in the first line treatment of the individual studies. Table S3–2. Efficacy of 7-day concomitant therapy in the first line treatment of the individual studies. Table S3–3. Efficacy of 10-day concomitant therapy in the first line treatment of the individual studies. Table S3–4. Efficacy of 14-day concomitant therapy in the first line treatment of the individual studies. Table S4. Efficacy of 10–14 day hybrid therapy in the first line treatment of the individual studies. Table S5–1. Efficacy of 7-day or less bismuth quadruple therapy in the first line treatment of the individual studies. Table S5–2. Efficacy of 10-day bismuth quadruple therapy in the first line treatment of the individual studies. Table S5–3. Efficacy of 14-day bismuth quadruple therapy in the first line treatment of the individual studies. Table S6. Efficacy of Levofloxacin triple therapy in the first line treatment of the individual studies. (DOCX 221 kb) [file 12929_2018_471_MOESM1_ESM.docx]

**Table S1-1. Efficacy of 7-day triple therapy in the first line treatment of the individual studies**

|  | **Eradication rate** | | |
| --- | --- | --- | --- |
|  | **Clarithromycin susceptible** | **Clarithromycin resistant** | **overall** |
| Ducons 1999[1] | 85.5% (77/90) | 20% (2/10) | 79% (79/100 |
| Lamouliatte 1999[2] | 86% (82/95) | 0% (0/6) | 81.1% (82/101) |
| Lind 1999[3] | 97% (111/116) | 100% (2/2) | 95.7% (113/118) |
| Pilotto 1999[4] | 87% (33/38) | 50% (1/2) | 85% (34/40) |
| Hoshiya 2000[5] | 83.1% (79/95) | 33.3% (4/12) | 77.5% (83/107) |
| Kihira 2000[6] | 96.3% (79/82) | 0% (0/3) | 92.6% (79/85) |
| Laine 2000[7] | 94.6% (88/93) | 23% (3/13) | 85.8% (91/106) |
| Poon 2000 | 95% (37/39) | 0% (0/5) | 84% (37/44) |
| Kalach 2001[8] | 100% (50/50) | 0% (0/11) | 81.9% (50/61) |
| Tankovic 2001[9] | 79% (67/85) | 12% (2/17) | 67.6% (69/102) |
| Bago 2002[10] | 89.7% (35/39) | 40% (2/5) | 84% (37/44) |
| Katelaris 2002[11] | 86% (36/42) | 25% (1/4) | 80.4% (37/46) |
| Murakami 2002[12] | 94% (226/241) | 4% (1/25) | 85.3% (227/266) |
| Peitz 2002[13] | 83% (10/12) | 30% (7/23) | 48.5% (17/35) |
| Bochenek 2003[14] | 77.3% (130/182) | 29.1% (7/24) | 66.5% (137/206) |
| Kawabata 2003[15] | 85.5% (130/152) | 23.8% (5/21) | 78% (135/173) |
| Miki 2003[16] | 97.5% (119/122) | 6.3% (1/16) | 87% (120/138) |
| Lamouliatte 2003[17] | 85.7% (18/21) | 8% (2/24) | 44.4%(20/45) |
| De Francesco 2006[18] | 86% (51/59) | 38.9% (7/18) | 75.3% (58/77) |
| Demir M 2009[19] | 70% (14/20) | 27.8% (10/36) | 42.9% (24/56) |
| Filipec Kanizaj 2009[20] | 79.5% (70/88) | 37.5% (3/8) | 76% (73/96) |
| Zheng 2010[21] | 62.1% (18/29) | 22.2% (2/9) | 52.6% (20/38) |
| Liou 2010[22] | 90.7% (107/118) | 44.4% (4/9) | 87.4% (111/127) |
| Malfertheiner 2011[23] | 85% (90/106) | 8% (2/25) | 70.2% (92/131) |
| Hsu 2014[24] | 90.6% (29/32) | 57.1% (4/7) | 84.61% (33/39) |
| Park 2014[25] | 94.1% (80/85) | 20% (3/15) | 83% (83/100) |
| Lee 2014[26] | 100% (7/7) | 0% (0/6) | 53.8% (7/13) |
| Tai 2015[27] | 100% (15/15) | 0% (0/19) | 44.1% (15/34) |
| Yang 2015[28] | 93.5% (116/124) | 19.2% (5/26) | 80.7% (121/150) |
| Tong 2015[29] | 91.4% (308/337) | 79.31% (23/29) | 90.4% (331/366) |
| Tepes 2016[30] | 89.7% (87/97) | 33.3% (4/12) | 83.5% (91/109) |
| Adachi 2017[31] | 87.9% (29/33) | 51.9% (14/27) | 71.7% (43/60) |
| **Total** | **88.5% (2428/2744)** | **25.8% (121/469)** | **79.33% (2549/3213)** |

**Table S1-2. Efficacy of 10-day triple therapy in the first line treatment of the individual studies**

|  | **Eradication rate** | | |
| --- | --- | --- | --- |
|  | **Clarithromycin susceptible** | **Clarithromycin resistant** | **overall** |
| Vaira 2007[32] | 94.5% (86/91) | 28.6% (6/21) | 82.1% (92/112) |
| Filipec Kanizaj 2009[20] | 90.4% (75/83) | 57.1% (4/7) | 87.8% (79/90) |
| Zhou 2014[33] | 75.9% (67/75) | 49.1% (26/53) | 72.7% (93/128) |
| Auesomwang 2018[34] | 86.7% (39/45) | 33.3% (1/3) | 83.3% (40/48) |
| **Total** | **90.8% (267/294)** | **44% (37/84)** | **80.4% (304/378)** |
|  |  |  |  |

**Table S1-3. Efficacy of 14-day triple therapy in the first line treatment of the individual studies**

|  | **Eradication rate** | | |
| --- | --- | --- | --- |
|  | **Clarithromycin susceptible** | **Clarithromycin resistant** | **overall** |
| Lehman 2000[35] | 87% (23/29) | 0% (0/3) | 72% (23/32) |
| McMahon 2003[36] | 87.5% (35/40) | 23% (3/13) | 71.6% (38/53) |
| Filipec Kanizaj 2009[20] | 97.5% (39/40) | 75% (3/4) | 95.5% (42/44) |
| Liou 2013[37] | 91% (137/151) | 55% (11/20) | 86.5% (148/171) |
| Alsohaibani 2015[38] | 74.1% (20/27) | 33.3% (1/3) | 70% (21/30) |
| Liou 2016[39] | 91.5% (335/366) | 44.4% (16/36) | 87.3% (351/402) |
| Liou 2016[40] | 88% (252/286) | 44% (21/48) | 81.7% (273/334) |
| **Total** | **89.6% (841/939)** | **43.3% (55/127)** | **84% (896/1066)** |
|  |  |  |  |

**Table S2-1. Efficacy of 10-day sequential therapy in the first line treatment of the individual studies**

|  | **Eradication rate** | | | | |
| --- | --- | --- | --- | --- | --- |
|  | **Clarithromycin susceptible** | | **Clarithromycin resistant** | | **overall** |
|  | **Met-S** | **Met-R** | **Met-S** | **Met-R** |  |
| De Francesco 2006[18] | 98% (51/59) | | 81.8% (18/22) | | 85.2% (69/81) |
| Vaira 2007[32] | 94.7% (108/114) | | 88.9% (8/9) | | 94.3% (116/123) |
| Romano 2010[41] | 88.5% (23/26) | 92.9% (13/14) | 100% (9/9) | 0% (0/3) | 86.5% (45/52) |
| Wu 2010[42] | 96.3% (53/55) | 95.4% (21/22) | 57.1% (4/7) | 33.3% (1/3) | 90.8% (79/87) |
| Huang 2012[43] | 93.8% (15/16) | 85.7% (12/14) | 100% (1/1) | 50% (2/4) | 85.7% (30/35) |
| Liou 2013[37] | 95% (123/129) | 78% (29/37) | 70% (7/10) | 43% (3/7) | 88.5% (162/183) |
| Hsu 2014[24] | 100% (28/28) | 90.9% (10/11) | 66.7% (2/3) | 50% (1/2) | 93.2% (41/44) |
| Zhou 2014[33] | 97% (32/33) | 87.8% (43/49) | 88.9% (8/9) | 43.9% (18/41) | 76.5% (101/132) |
| Lee 2014[26] | 100% (6/6) | 0% (0/1) | 0% (0/2) | 25% (1/4) | 53.8% (7/13) |
| Yang 2015[28] | 94% (79/84) | 87.8% (36/41) | 81.8% (9/11) | 28.6% (4/14) | 85.3% (128/150) |
| Tsay 2015[44] | 90.7% (39/43) | | 100% (7/7) | | 92% (46/50) |
| Alsohaibani 2015[38] | 70.1% (17/24) | | 10% (1/10) | | 52.9% (18/34) |
| Tepes 2016[30] | 97.2% (104/107) | | 66.7% (6/9) | | 94.8% (110/116) |
| Georgopoulos 2016[45] | 92% (62/67) | 79.5% (31/39) | 83.9% (26/31) | 36.4% (4/11) | 83.1% (123/148) |
| Liou 2016[39] | 91.1% (257/282) | 89.2% (74/83) | 70.4% (19/27) | 50% (9/18) | 87.6% (359/410) |
| Kim 2017[46] | 82.5% (52/63) | | 53.9% (7/13) | | 77.6% (59/76) |
| Fiorini 2018[47] | 91% (96/105) | 93% (26/28) | 80% (16/20) | 85% (28/33) | 89.2% (166/186) |
| Auesomwang 2018[34] | 83.3% (30/36) | | 100% (6/6) | | 85.7% (36/42) |
| **Subtotal** | **93.1% (774/831)** | **87% (295/339)** | **77.7% (101/130)** | **44.9% (79/176)** | **84.6% (1249/1476)** |
| **Total** | **91% (1470/1616)** | | **65% (225/346)** | | **86.4% (1695/1962)** |
|  |  | |  | |  |

**Table S2-2. Efficacy of 14-day sequential therapy in the first line treatment of the individual studies**

|  | **Eradication rate** | | | | |
| --- | --- | --- | --- | --- | --- |
|  | **Clarithromycin susceptible** | | **Clarithromycin resistant** | | **overall** |
|  | **Met-S** | **Met-R** | **Met-S** | **Met-R** |  |
| Liou 2013[37] | 99% (116/117) | 91% (30/33) | 71% (10/14) | 0% (0/1) | 94.5% (156/165) |
| Liou 2018[48] | 99.2% (122/123) | 97.3% (36/37) | 78.6% (11/14) | 71.4% (5/7) | 96.1% (174/181) |
| **Subtotal** | **99.2% (238/240)** | **94.3% (66/70)** | **75% (21/28)** | **62.5% (5/8)** |  |
| **Total** | **98.1% (304/310)** | | **72.2% (26/36)** | | **95.4% (330/346)** |
|  |  | |  | |  |

**Table S3-1. Efficacy of 5-day or less concomitant therapy in the first line treatment of the individual studies**

|  | **Eradication rate** | | | | |
| --- | --- | --- | --- | --- | --- |
|  | **Clarithromycin susceptible** | | **Clarithromycin resistant** | | **overall** |
|  | **Met-S** | **Met-R** | **Met-S** | **Met-R** |  |
| Neville 1999[49] | 94.7%(18/19) | 85% (17/20) |  |  |  |
| Treiber 2002[50] | 89·7% (35/39) | 50% (6/12) | - | 50% (2/4) |  |
| **Subotal** | **91.4% (53/58)** | **71.9% (23/32)** | **-** | **50% (2/4)** | **83% (78/94)** |
| **Total** | **84.4% (76/90)** | | **50% (2/4)** | | **83% (78/94)** |
|  |  | |  | |  |

**Table S3-2. Efficacy of 7-day concomitant therapy in the first line treatment of the individual studies**

|  | **Eradication rate** | | | | |
| --- | --- | --- | --- | --- | --- |
|  | **Clarithromycin susceptible** | | **Clarithromycin resistant** | | **overall** |
|  | **Met-S** | **Met-R** | **Met-S** | **Met-R** |  |
| Okada 1999[51] | 95·2% (118/124) | 95·7% (22/23) | 100% (12/12) | 75% (3/4) | 95.1% (155/163) |
| Hsu 2014[24] | 100% (23/23) | 100% (18/18) | 100% (2/2) | 66·7% (2/3) | 97.8% (45/46) |
| Tai 2015[27] | - | - | - | 33.3% (1/3) | - |
| **Subtotal** | **95.9% (141/147)** | **97.6% (40/41)** | **100% (14/14)** | **60% (6/10)** | **94.8% (201/212)** |
| **Total** | **96.3% (181/188)** | | **83.3% (20/24)** | | **94.8% (201/212)** |
|  |  | |  | |  |

**Table S3-3. Efficacy of 10-day concomitant therapy in the first line treatment of the individual studies**

|  | **Eradication rate** | | | | |
| --- | --- | --- | --- | --- | --- |
|  | **Clarithromycin susceptible** | | **Clarithromycin resistant** | | **overall** |
|  | **Met-S** | **Met-R** | **Met-S** | **Met-R** |  |
| Wu 2010[42] | 91·2% (52/57) | 95·5% (21/22) | - | 75% (3/4) | 91.6% (76/83) |
| Molina–Infante 2012[52] | 89% (25/28) | 100% (8/8) | 100% (5/5) | 75% (3/4) | 91.1% (41/45) |
| Huang 2012[43] | 90·9% (20/22) | 100% (14/14) | 100% (1/1) | 100% (2/2) | 94.9% (37/39) |
| Georgopoulos 2013[53] | 100% (30/30) | 100% (25/25) | 87% (13/15) | 70% (7/10) | 93.7% (75/80) |
| Georgopoulos 2016[45] | 97·1% (67/69) | 97·4% (38/39) | 89·3% (25/28) | 75% (9/12) | 93.9% (139/148) |
| Liou 2016[40] | 95·3% (183/192) | 95·2% (59/62) | 76·5% (26/34) | 63·2% (12/19) | 91.2% (280/307) |
| Kim 2017[46] | 86.2% (56/65) | | 93.3% (14/15) | | 87.5% (70/80) |
| **subtotal** | **94.7% (377/398)** | **97.1% (165/170)** | **84.3% (70/83)** | **70.6% (36/51)** | **92.3% (648/702)** |
| **Total** | **94.5% (598/633)** | | **80.5% (120/149)** | | **91.8% (718/782)** |

**Table S3-4. Efficacy of 14-day concomitant therapy in the first line treatment of the individual studies**

|  | **Eradication rate** | | | | |
| --- | --- | --- | --- | --- | --- |
|  | **Clarithromycin susceptible** | | **Clarithromycin resistant** | | **overall** |
|  | **Met-S** | **Met-R** | **Met-S** | **Met-R** |  |
| Molina–Infante 2013[54] | 94% (17/18) | 100% (8/8) | 100% (5/5) | 100% (3/3) |  |
| **Total** | **96.1% (25/26)** | | **100% (8/8)** | | **97.1% (33/34)** |
|  |  | |  | |  |

**Table S4. Efficacy of 10-14 day hybrid therapy in the first line treatment of the individual studies**

|  | **Eradication rate** | | | | |
| --- | --- | --- | --- | --- | --- |
|  | **Clarithromycin susceptible** | | **Clarithromycin resistant** | | **overall** |
|  | **Met-S** | **Met-R** | **Met-S** | **Met-R** |  |
| Hsu 2011[55] | 100%(25/25) | 100%(28/28) | 0 | 100%(4/4) | 100% (57/57) |
| Molina-Infante 2013[52] | 100%(18/18) | 87.5%(7/8) | 100%(5/5) | 33%(1/3) | 91.2% (31/34) |
| Hsu 2015 *[56] | 99% (74/75) | | 90% (9/10) | | 97.6% (83/85) |
| Song 2016[57] | 91.7% (66/72) | | 74.6% (47/63) | | 83.7% (113/135) |
| Tsay 2017[58] | 100% (28/28) | | 83.3% (5/6) | | 97.1% (33/34) |
| Georgopoulos 2018[59] | 94.3% (83/88) | | 91.4% (31/35) | | 92.7% (114/123) |
| Hsu 2018 **[60] | 98.5% (65/66) | 100% (24/24) | 90.9% (10/11) | 83.3% (5/6) | 97.2% (104/107) |
| **Subtotal** | **99.1% (108/109)** | **98.3% (59/60)** | **93.7% (15/16)** | **76.9% (10/13)** |  |
| **Total** | **96.8% (418/432)** | | **81.8% (117/143)** | | **93% (535/575)** |

* Reverse hybrid 12 days ** Reverse hybrid 14 days

**Table S5-1. Efficacy of 7-day or less bismuth quadruple therapy in the first line treatment of the individual studies**

|  | **Eradication rate** | | | | |
| --- | --- | --- | --- | --- | --- |
|  | **Metronidazole susceptible** | | **Metronidazole resistant** | | **overall** |
|  | **CLA-S** | **CLA-R** | **CLA-S** | **CLA-R** |  |
| de Boer WA 1995*[61] | 95% (38/40) | | 33.3% (1/3) | | 90.7% (39/43) |
| de Boer WA 1996*[62] | 100% (12/12) | | 100% (2/2) | | 100% (14/14) |
| de Boer WA 1998*[63] | 93.2% (41/44) | | 62.5% (5/8) | | 88.5% (46/52) |
| Lahaie 2001[64] | 89.5% (51/57) | | 70.8% (17/24) | | 84% (68/81) |
| Katelaris 2002[11] | 80% (16/20) | | 81% (17/21) | | 80.5% (33/41) |
| de Boer SY 2003*[65] | 91% (42/46) | | 57.1% (4/7) | | 86.8% (46/53) |
| de Boer SY 2003[65] | 97% (32/33) | | 100% (7/7) | | 97.5% (39/40) |
| Vilaichone 2015[66] | 90.1% (20/22) | | 72.7% (16/22) | | 81.8% (36/44) |
| **Total** | **92% (252/274)** | | **73.4% (69/94)** | | **87.2% (321/368)** |

* 4-day regimen

**Table S5-2. Efficacy of 10-day bismuth quadruple therapy in the first line treatment of the individual studies**

|  | **Eradication rate** | | | | |
| --- | --- | --- | --- | --- | --- |
|  | **Metronidazole susceptible** | | **Metronidazole resistant** | | **overall** |
|  | **CLA-S** | **CLA-R** | **CLA-S** | **CLA-R** |  |
| Graham 1997[67] | 89.7% (26/29) | | 41.2% (7/17) | | 71.7% (33/46) |
| O'Morain 2003[68] | 99% (75/76) | | 95% (38/40) | | 97.4% (113/116) |
| Laine 2003[69] | 95.2% (60/63) | | 86.7% (39/45) | | 91.7% (99/108) |
| Zheng 2010[70] | 92.3% (24/26) | | 92.3% (12/13) | | 92.3% (36/39) |
| Malfertheiner 2011[23] | 95% (98/103) | | 91% (38/42) | | 93.8% (136/145) |
| Liou 2016[40] | 92% (180/196) | 89% (33/37) | 93% (71/76) | 94% (16/17) | 92% (300/326) |
| Fiorini 2018[47] | 93% (84/90) | 97% (32/33) | 93% (27/29) | 85% (34/40) | 92.2% (177/192) |
| Liou 2018[48] | 96.7% (118/122) | 100% (17/17) | 100% (32/32) | 87.5% (7/8) | 97.2% (174/179) |
| Xie 2018[71] | 94.4% (17/18) | | 91.6% (76/83) | | 92.1% (93/101) |
| **Subtotal** | **93.6% (382/408)** | **94.2% (82/87)** | **94.9% (130/137)** | **87.7% (57/65)** | **93.4% (651/697)** |
| **Total** | **94.3% (764/810)** | | **89.8% (397/442)** | | **92.7% (1161/1252)** |

**Table S5-3. Efficacy of 14-day bismuth quadruple therapy in the first line treatment of the individual studies**

|  | **Eradication rate** | | | | |
| --- | --- | --- | --- | --- | --- |
|  | **Metronidazole susceptible** | | **Metronidazole resistant** | | **overall** |
|  | **CLA-S** | **CLA-R** | **CLA-S** | **CLA-R** |  |
| Graham 2004[72] | 96% (24/25) | | 83.3% (10/12) | | 91.9% (34/37) |
| Hsu 2018[60] | 95.8% (68/71) | 100% (7/7) | 100% (26/26) | 83.3% (5/6) | 96.4% (106/110) |
| **Total** | **96.1% (99/103)** | | **93.2% (41/44)** | | **95.2% (140/147)** |

**Table S6. Efficacy of Levofloxacin triple therapy in the first line treatment of the individual studies**

|  | **Eradication rate** | | |
| --- | --- | --- | --- |
|  | **Levofloxacin susceptible** | **Levofloxacin resistant** | **overall** |
| Liou 2010[22] | 84.4% (103/122) | 50% (5/10) | 81.8% (108/132) |
| Liao 2013*[73] | 97.3% (37/38) | 37.5% (6/16) | 79.6% (43/54) |
| **Total** | **87.5% (140/160)** | **42.3% (11/26)** | **81.2% (151/186)** |

* Levofloxacin 500mg qd x 14 days

**Reference**

1. Gomollon F, Ducons JA, Ferrero M, Garcia Cabezudo J, Guirao R, Simon MA, et al. Quadruple therapy is effective for eradicating Helicobacter pylori after failure of triple proton-pump inhibitor-based therapy: a detailed, prospective analysis of 21 consecutive cases. Helicobacter. 1999;4(4):222-5.

2. Lamouliatte H, Samoyeau R, De Mascarel A and Megraud F. Double vs. single dose of pantoprazole in combination with clarithromycin and amoxycillin for 7 days, in eradication of Helicobacter pylori in patients with non-ulcer dyspepsia. Aliment Pharmacol Ther. 1999;13(11):1523-30.

3. Lind T, Megraud F, Unge P, Bayerdorffer E, O'Morain C, Spiller R, et al. The MACH2 study: role of omeprazole in eradication of Helicobacter pylori with 1-week triple therapies. Gastroenterology. 1999;116(2):248-53.

4. Pilotto A, Franceschi M, Leandro G, Bozzola L, Fortunato A, Rassu M, et al. Efficacy of 7 day lansoprazole-based triple therapy for Helicobacter pylori infection in elderly patients. J Gastroenterol Hepatol. 1999;14(5):468-75.

5. Hoshiya S, Watanabe K, Tokunaga K, Tanaka A, Ninomiya H, Shingaki M, et al. Relationship between eradication therapy and clarithromycin-resistant Helicobacter pylori in Japan. J Gastroenterol. 2000;35(1):10-4.

6. Kihira K, Satoh K, Saifuku K, Kawakami S, Fukazawa K, Ishino Y, et al. Rabeprazole, amoxycillin and low- or high-dose clarithromycin for cure of Helicobacter pylori infection. Aliment Pharmacol Ther. 2000;14(8):1083-7.

7. Laine L, Fennerty MB, Osato M, Sugg J, Suchower L, Probst P, et al. Esomeprazole-based Helicobacter pylori eradication therapy and the effect of antibiotic resistance: results of three US multicenter, double-blind trials. Am J Gastroenterol. 2000;95(12):3393-8.

8. Kalach N, Benhamou PH, Campeotto F, Bergeret M, Dupont C and Raymond J. Clarithromycin resistance and eradication of Helicobacter pylori in children. Antimicrob Agents Chemother. 2001;45(7):2134-5.

9. Tankovic J, Lamarque D, Lascols C, Soussy CJ and Delchier JC. Impact of Helicobacter pylori resistance to clarithromycin on the efficacy of the omeprazole-amoxicillin-clarithromycin therapy. Aliment Pharmacol Ther. 2001;15(5):707-13.

10. Bago J, Strinic D, Halle ZB, Jandric D, Tomic M, Bilic A, et al. Effect of Helicobacter pylori eradication on extent of duodenal gastric metaplasia and grade of gastritis. Coll Antropol. 2002;26(2):557-63.

11. Katelaris PH, Forbes GM, Talley NJ and Crotty B. A randomized comparison of quadruple and triple therapies for Helicobacter pylori eradication: The QUADRATE Study. Gastroenterology. 2002;123(6):1763-9.

12. Murakami K, Sato R, Okimoto T, Nasu M, Fujioka T, Kodama M, et al. Eradication rates of clarithromycin-resistant Helicobacter pylori using either rabeprazole or lansoprazole plus amoxicillin and clarithromycin. Aliment Pharmacol Ther. 2002;16(11):1933-8.

13. Peitz U, Sulliga M, Wolle K, Leodolter A, Von Arnim U, Kahl S, et al. High rate of post-therapeutic resistance after failure of macrolide-nitroimidazole triple therapy to cure Helicobacter pylori infection: impact of two second-line therapies in a randomized study. Aliment Pharmacol Ther. 2002;16(2):315-24.

14. Bochenek WJ, Peters S, Fraga PD, Wang W, Mack ME, Osato MS, et al. Eradication of Helicobacter pylori by 7-day triple-therapy regimens combining pantoprazole with clarithromycin, metronidazole, or amoxicillin in patients with peptic ulcer disease: results of two double-blind, randomized studies. Helicobacter. 2003;8(6):626-42.

15. Kawabata H, Habu Y, Tomioka H, Kutsumi H, Kobayashi M, Oyasu K, et al. Effect of different proton pump inhibitors, differences in CYP2C19 genotype and antibiotic resistance on the eradication rate of Helicobacter pylori infection by a 1-week regimen of proton pump inhibitor, amoxicillin and clarithromycin. Aliment Pharmacol Ther. 2003;17(2):259-64.

16. Miki I, Aoyama N, Sakai T, Shirasaka D, Wambura CM, Maekawa S, et al. Impact of clarithromycin resistance and CYP2C19 genetic polymorphism on treatment efficacy of Helicobacter pylori infection with lansoprazole- or rabeprazole-based triple therapy in Japan. Eur J Gastroenterol Hepatol. 2003;15(1):27-33.

17. Lamouliatte H, Megraud F, Delchier JC, Bretagne JF, Courillon-Mallet A, De Korwin JD, et al. Second-line treatment for failure to eradicate Helicobacter pylori: a randomized trial comparing four treatment strategies. Aliment Pharmacol Ther. 2003;18(8):791-7.

18. De Francesco V, Margiotta M, Zullo A, Hassan C, Troiani L, Burattini O, et al. Clarithromycin-resistant genotypes and eradication of Helicobacter pylori. Ann Intern Med. 2006;144(2):94-100.

19. Demir M, Gokturk HS, Ozturk NA, Arslan H, Serin E and Yilmaz U. Clarithromycin resistance and efficacy of clarithromycin-containing triple eradication therapy for Helicobacter pylori infection in type 2 diabetes mellitus patients. South Med J. 2009;102(11):1116-20.

20. Filipec Kanizaj T, Katicic M, Skurla B, Ticak M, Plecko V and Kalenic S. Helicobacter pylori eradication therapy success regarding different treatment period based on clarithromycin or metronidazole triple-therapy regimens. Helicobacter. 2009;14(1):29-35.

21. Zheng Q, Chen WJ, Lu H, Sun QJ and Xiao SD. Comparison of the efficacy of triple versus quadruple therapy on the eradication of Helicobacter pylori and antibiotic resistance. J Dig Dis. 2010;11(5):313-8.

22. Liou JM, Lin JT, Chang CY, Chen MJ, Cheng TY, Lee YC, et al. Levofloxacin-based and clarithromycin-based triple therapies as first-line and second-line treatments for Helicobacter pylori infection: a randomised comparative trial with crossover design. Gut. 2010;59(5):572-8.

23. Malfertheiner P, Bazzoli F, Delchier JC, Celinski K, Giguere M, Riviere M, et al. Helicobacter pylori eradication with a capsule containing bismuth subcitrate potassium, metronidazole, and tetracycline given with omeprazole versus clarithromycin-based triple therapy: a randomised, open-label, non-inferiority, phase 3 trial. Lancet. 2011;377(9769):905-13.

24. Hsu PI, Wu DC, Chen WC, Tseng HH, Yu HC, Wang HM, et al. Randomized controlled trial comparing 7-day triple, 10-day sequential, and 7-day concomitant therapies for Helicobacter pylori infection. Antimicrob Agents Chemother. 2014;58(10):5936-42.

25. Park CS, Lee SM, Park CH, Koh HR, Jun CH, Park SY, et al. Pretreatment antimicrobial susceptibility-guided vs. clarithromycin-based triple therapy for Helicobacter pylori eradication in a region with high rates of multiple drug resistance. Am J Gastroenterol. 2014;109(10):1595-602.

26. Lee JW, Kim N, Kim JM, Nam RH, Kim JY, Lee JY, et al. A comparison between 15-day sequential, 10-day sequential and proton pump inhibitor-based triple therapy for Helicobacter pylori infection in Korea. Scand J Gastroenterol. 2014;49(8):917-24.

27. Tai WC, Liang CM, Lee CH, Chiu CH, Hu ML, Lu LS, et al. Seven-Day Nonbismuth Containing Quadruple Therapy Could Achieve a Grade "A" Success Rate for First-Line Helicobacter pylori Eradication. Biomed Res Int. 2015;2015:623732.

28. Yang JC, Lin CJ, Wang HL, Chen JD, Kao JY, Shun CT, et al. High-dose dual therapy is superior to standard first-line or rescue therapy for Helicobacter pylori infection. Clin Gastroenterol Hepatol. 2015;13(5):895-905 e5.

29. Tong YF, Lv J, Ying LY, Xu F, Qin B, Chen MT, et al. Seven-day triple therapy is a better choice for Helicobacter pylori eradication in regions with low antibiotic resistance. World J Gastroenterol. 2015;21(46):13073-9.

30. Tepes B, Vujasinovic M, Seruga M, Stefanovic M, Forte A and Jeverica S. Randomized clinical trial comparing 10-day sequential, 7-day concomitant and 7-day standard triple therapies for Helicobacter pylori eradication. Eur J Gastroenterol Hepatol. 2016;28(6):676-83.

31. Adachi T, Matsui S, Watanabe T, Okamoto K, Okamoto A, Kono M, et al. Comparative Study of Clarithromycin- versus Metronidazole-Based Triple Therapy as First-Line Eradication for Helicobacter pylori. Oncology. 2017;93 Suppl 1:15-9.

32. Vaira D, Zullo A, Vakil N, Gatta L, Ricci C, Perna F, et al. Sequential therapy versus standard triple-drug therapy for Helicobacter pylori eradication: a randomized trial. Ann Intern Med. 2007;146(8):556-63.

33. Zhou L, Zhang J, Chen M, Hou X, Li Z, Song Z, et al. A comparative study of sequential therapy and standard triple therapy for Helicobacter pylori infection: a randomized multicenter trial. Am J Gastroenterol. 2014;109(4):535-41.

34. Auesomwang C, Maneerattanaporn M, Chey WD, Kiratisin P, Leelakusolwong S and Tanwandee T. Ten-day high-dose proton pump inhibitor triple therapy versus sequential therapy for Helicobacter pylori eradication. J Gastroenterol Hepatol. 2018.

35. Lehmann FS, Drewe J, Terracciano L and Beglinger C. Effect of ornidazole and clarithromycin resistance on eradication of Helicobacter pylori in peptic ulcer disease. Aliment Pharmacol Ther. 2000;14(3):305-9.

36. McMahon BJ, Hennessy TW, Bensler JM, Bruden DL, Parkinson AJ, Morris JM, et al. The relationship among previous antimicrobial use, antimicrobial resistance, and treatment outcomes for Helicobacter pylori infections. Ann Intern Med. 2003;139(6):463-9.

37. Liou JM, Chen CC, Chen MJ, Chen CC, Chang CY, Fang YJ, et al. Sequential versus triple therapy for the first-line treatment of Helicobacter pylori: a multicentre, open-label, randomised trial. Lancet. 2013;381(9862):205-13.

38. Alsohaibani F, Al Ashgar H, Al Kahtani K, Kagevi I, Peedikayil M, Alfadda A, et al. Prospective trial in Saudi Arabia comparing the 14-day standard triple therapy with the 10-day sequential therapy for treatment of Helicobacter pylori infection. Saudi J Gastroenterol. 2015;21(4):220-5.

39. Liou JM, Chen CC, Chang CY, Chen MJ, Chen CC, Fang YJ, et al. Sequential therapy for 10 days versus triple therapy for 14 days in the eradication of Helicobacter pylori in the community and hospital populations: a randomised trial. Gut. 2016;65(11):1784-92.

40. Liou JM, Fang YJ, Chen CC, Bair MJ, Chang CY, Lee YC, et al. Concomitant, bismuth quadruple, and 14-day triple therapy in the first-line treatment of Helicobacter pylori: a multicentre, open-label, randomised trial. Lancet. 2016;388(10058):2355-65.

41. Romano M, Cuomo A, Gravina AG, Miranda A, Iovene MR, Tiso A, et al. Empirical levofloxacin-containing versus clarithromycin-containing sequential therapy for Helicobacter pylori eradication: a randomised trial. Gut. 2010;59(11):1465-70.

42. Wu DC, Hsu PI, Wu JY, Opekun AR, Kuo CH, Wu IC, et al. Sequential and concomitant therapy with four drugs is equally effective for eradication of H pylori infection. Clin Gastroenterol Hepatol. 2010;8(1):36-41 e1.

43. Huang YK, Wu MC, Wang SS, Kuo CH, Lee YC, Chang LL, et al. Lansoprazole-based sequential and concomitant therapy for the first-line Helicobacter pylori eradication. J Dig Dis. 2012;13(4):232-8.

44. Tsay FW, Wu DC, Kao SS, Tsai TJ, Lai KH, Cheng JS, et al. Reverse sequential therapy achieves a similar eradication rate as standard sequential therapy for Helicobacter pylori eradication: a randomized controlled trial. Helicobacter. 2015;20(1):71-7.

45. Georgopoulos SD, Xirouchakis E, Martinez-Gonzales B, Zampeli E, Grivas E, Spiliadi C, et al. Randomized clinical trial comparing ten day concomitant and sequential therapies for Helicobacter pylori eradication in a high clarithromycin resistance area. Eur J Intern Med. 2016;32:84-90.

46. Kim SY, Lee SW, Choe JW, Jung SW, Hyun JJ, Jung YK, et al. Helicobacter pylori eradication rates of concomitant and sequential therapies in Korea. Helicobacter. 2017;22(6).

47. Fiorini G, Zullo A, Saracino IM, Gatta L, Pavoni M and Vaira D. Pylera and sequential therapy for first-line Helicobacter pylori eradication: a culture-based study in real clinical practice. Eur J Gastroenterol Hepatol. 2018;30(6):621-5.

48. Liou JM, Chen CC, Fang YJ, Chen PY, Chang CY, Chou CK, et al. 14 day sequential therapy versus 10 day bismuth quadruple therapy containing high-dose esomeprazole in the first-line and second-line treatment of Helicobacter pylori: a multicentre, non-inferiority, randomized trial. J Antimicrob Chemother. 2018.

49. Neville PM, Everett S, Langworthy H, Tompkins D, Mapstone NP, Axon AT, et al. The optimal antibiotic combination in a 5-day Helicobacter pylori eradication regimen. Aliment Pharmacol Ther. 1999;13(4):497-501.

50. Treiber G, Wittig J, Ammon S, Walker S, van Doorn LJ and Klotz U. Clinical outcome and influencing factors of a new short-term quadruple therapy for Helicobacter pylori eradication: a randomized controlled trial (MACLOR study). Arch Intern Med. 2002;162(2):153-60.

51. Okada M, Nishimura H, Kawashima M, Okabe N, Maeda K, Seo M, et al. A new quadruple therapy for Helicobacter pylori: influence of resistant strains on treatment outcome. Aliment Pharmacol Ther. 1999;13(6):769-74.

52. Molina-Infante J, Pazos-Pacheco C, Vinagre-Rodriguez G, Perez-Gallardo B, Duenas-Sadornil C, Hernandez-Alonso M, et al. Nonbismuth quadruple (concomitant) therapy: empirical and tailored efficacy versus standard triple therapy for clarithromycin-susceptible Helicobacter pylori and versus sequential therapy for clarithromycin-resistant strains. Helicobacter. 2012;17(4):269-76.

53. Georgopoulos S, Papastergiou V, Xirouchakis E, Laoudi F, Lisgos P, Spiliadi C, et al. Nonbismuth quadruple "concomitant" therapy versus standard triple therapy, both of the duration of 10 days, for first-line H. pylori eradication: a randomized trial. J Clin Gastroenterol. 2013;47(3):228-32.

54. Molina-Infante J, Romano M, Fernandez-Bermejo M, Federico A, Gravina AG, Pozzati L, et al. Optimized nonbismuth quadruple therapies cure most patients with Helicobacter pylori infection in populations with high rates of antibiotic resistance. Gastroenterology. 2013;145(1):121-8 e1.

55. Hsu PI, Wu DC, Wu JY and Graham DY. Modified sequential Helicobacter pylori therapy: proton pump inhibitor and amoxicillin for 14 days with clarithromycin and metronidazole added as a quadruple (hybrid) therapy for the final 7 days. Helicobacter. 2011;16(2):139-45.

56. Hsu PI, Kao SS, Wu DC, Chen WC, Peng NJ, Yu HC, et al. A Randomized Controlled Study Comparing Reverse Hybrid Therapy and Standard Triple Therapy for Helicobacter pylori Infection. Medicine (Baltimore). 2015;94(48):e2104.

57. Song Z, Zhou L, Zhang J, He L, Bai P and Xue Y. Hybrid Therapy as First-Line Regimen for Helicobacter pylori Eradication in Populations with High Antibiotic Resistance Rates. Helicobacter. 2016;21(5):382-8.

58. Tsay FW, Wu DC, Yu HC, Kao SS, Lin KH, Cheng JS, et al. A Randomized Controlled Trial Shows that both 14-Day Hybrid and Bismuth Quadruple Therapies Cure Most Patients with Helicobacter pylori Infection in Populations with Moderate Antibiotic Resistance. Antimicrob Agents Chemother. 2017;61(11).

59. Georgopoulos SD, Papastergiou V, Martinez-Gonzalez B, Xirouchakis E, Familias I, Sgouras D, et al. Hybrid therapy as first-line regimen for Helicobacter pylori eradication in a high clarithromycin resistance area: a prospective open-label trial. Ann Gastroenterol. 2018;31(2):205-10.

60. Hsu PI, Tsay FW, Graham DY, Tsai TJ, Tsai KW, Kao JY, et al. Equivalent Efficacies of Reverse Hybrid and Bismuth Quadruple Therapies in Eradication of Helicobacter pylori Infection in a Randomized Controlled Trial. Clin Gastroenterol Hepatol. 2018. Mar 31. pii: S1542-3565(18)30328-8. doi: 10.1016/j.cgh.2018.03.031. [Epub ahead of print]

61. De Boer WA, Driessen WM and Tytgat GN. Only four days of quadruple therapy can effectively cure Helicobacter pylori infection. Aliment Pharmacol Ther. 1995;9(6):633-8.

62. de Boer WA, van Etten RJ, Schade RW, Ouwehand ME, Schneeberger PM and Tytgat GN. 4-day lansoprazole quadruple therapy: a highly effective cure for Helicobacter pylori infection. Am J Gastroenterol. 1996;91(9):1778-82.

63. de Boer WA, van Etten RJ and Schneeberger PM. Four-day lansoprazole-quadruple therapy in the routine treatment of Helicobacter pylori infection. Neth J Med. 1998;52(1):10-5.

64. Lahaie R, Farley A, Dallaire C, Archambault A, Fallone CA, Ponich T, et al. Bismuth-based quadruple therapy with bismuth subcitrate, metronidazole, tetracycline and omeprazole in the eradication of Helicobacter pylori. Can J Gastroenterol. 2001;15(9):581-5.

65. de Boer SY, v d Meeberg PC, Siem H and de Boer WA. Comparison of four-day and seven-day pantoprazole-based quadruple therapy as a routine treatment for Helicobacter pylori infection. Neth J Med. 2003;61(6):218-22.

66. Vilaichone RK, Prapitpaiboon H, Gamnarai P, Namtanee J, Wongcha-um A, Chaithongrat S, et al. Seven-Day Bismuth-based Quadruple Therapy as an Initial Treatment for Helicobacter pylori Infection in a High Metronidazole Resistant Area. Asian Pac J Cancer Prev. 2015;16(14):6089-92.

67. Graham DY, Hoffman J, el-Zimaity HM, Graham DP and Osato M. Twice a day quadruple therapy (bismuth subsalicylate, tetracycline, metronidazole plus lansoprazole) for treatment of Helicobacter pylori infection. Aliment Pharmacol Ther. 1997;11(5):935-8.

68. O'Morain C, Borody T, Farley A, De Boer WA, Dallaire C, Schuman R, et al. Efficacy and safety of single-triple capsules of bismuth biskalcitrate, metronidazole and tetracycline, given with omeprazole, for the eradication of Helicobacter pylori: an international multicentre study. Aliment Pharmacol Ther. 2003;17(3):415-20.

69. Laine L, Hunt R, El-Zimaity H, Nguyen B, Osato M and Spenard J. Bismuth-based quadruple therapy using a single capsule of bismuth biskalcitrate, metronidazole, and tetracycline given with omeprazole versus omeprazole, amoxicillin, and clarithromycin for eradication of Helicobacter pylori in duodenal ulcer patients: a prospective, randomized, multicenter, North American trial. Am J Gastroenterol. 2003;98(3):562-7.

70. Sun Q, Liang X, Zheng Q, Liu W, Xiao S, Gu W, et al. High efficacy of 14-day triple therapy-based, bismuth-containing quadruple therapy for initial Helicobacter pylori eradication. Helicobacter. 2010;15(3):233-8.

71. Xie Y, Pan X, Li Y, Wang H, Du Y, Xu J, et al. New single capsule of bismuth, metronidazole and tetracycline given with omeprazole versus quadruple therapy consisting of bismuth, omeprazole, amoxicillin and clarithromycin for eradication of Helicobacter pylori in duodenal ulcer patients: a Chinese prospective, randomized, multicentre trial. J Antimicrob Chemother. 2018;73(6):1681-7.

72. Graham DY, Belson G, Abudayyeh S, Osato MS, Dore MP and El-Zimaity HM. Twice daily (mid-day and evening) quadruple therapy for H. pylori infection in the United States. Dig Liver Dis. 2004;36(6):384-7.

73. Liao J, Zheng Q, Liang X, Zhang W, Sun Q, Liu W, et al. Effect of fluoroquinolone resistance on 14-day levofloxacin triple and triple plus bismuth quadruple therapy. Helicobacter. 2013;18(5):373-7.
